# MEMS Technology in Cardiology: Advancements and Applications in Heart Failure Management Focusing on the CardioMEMS Device

**DOI:** 10.3390/s24092922

**Published:** 2024-05-03

**Authors:** Francesco Ciotola, Stylianos Pyxaras, Harald Rittger, Veronica Buia

**Affiliations:** Medizinische Klinik I, Klinikum Fürth, Academic Teaching Hospital of the Friedrich-Alexander-University Erlangen-Nürnberg, Jakob-Henle Str. 1, 90766 Fürth, Germany; francesco.ciotola@klinikum-fuerth.de (F.C.); stylianos.pyxaras@klinikum-fuerth.de (S.P.); harald.rittger@klinikum-fuerth.de (H.R.)

**Keywords:** CardioMEMS, congestive heart failure (CHF), pulmonary artery hypertension (PAH), remote monitoring, telemedicine, microelectromechanical sensors

## Abstract

Heart failure (HF) is a complex clinical syndrome associated with significant morbidity, mortality, and healthcare costs. It is characterized by various structural and/or functional abnormalities of the heart, resulting in elevated intracardiac pressure and/or inadequate cardiac output at rest and/or during exercise. These dysfunctions can originate from a variety of conditions, including coronary artery disease, hypertension, cardiomyopathies, heart valve disorders, arrhythmias, and other lifestyle or systemic factors. Identifying the underlying cause is crucial for detecting reversible or treatable forms of HF. Recent epidemiological studies indicate that there has not been an increase in the incidence of the disease. Instead, patients seem to experience a chronic trajectory marked by frequent hospitalizations and stagnant mortality rates. Managing these patients requires a multidisciplinary approach that focuses on preventing disease progression, controlling symptoms, and preventing acute decompensations. In the outpatient setting, patient self-care plays a vital role in achieving these goals. This involves implementing necessary lifestyle changes and promptly recognizing symptoms/signs such as dyspnea, lower limb edema, or unexpected weight gain over a few days, to alert the healthcare team for evaluation of medication adjustments. Traditional methods of HF monitoring, such as symptom assessment and periodic clinic visits, may not capture subtle changes in hemodynamics. Sensor-based technologies offer a promising solution for remote monitoring of HF patients, enabling early detection of fluid overload and optimization of medical therapy. In this review, we provide an overview of the CardioMEMS device, a novel sensor-based system for pulmonary artery pressure monitoring in HF patients. We discuss the technical aspects, clinical evidence, and future directions of CardioMEMS in HF management.

## 1. Introduction

Heart failure (HF) affects millions of people worldwide and poses a substantial burden on healthcare systems [1,2,3]. The global occurrence of chronic HF is approximated to affect 1–2% of the adult population and is on the rise, which is primarily attributed to the aging demographic and advancements in cardiovascular disease management [4,5,6]. Although significant advancements have been achieved in treating heart failure, these patients still require close ambulatory follow-up and are frequently admitted to hospitals due to acute decompensation [7]. On top of this, previous research indicates that recurrent hospitalizations for decompensated heart failure are linked to deterioration in myocardial and renal function, as well as poorer survival outcomes [3,8,9]. In recent years, the landscape of HF management has witnessed a significant paradigm shift with the advent of remote monitoring techniques aimed at early detection of worsening disease [10,11]. This shift has been particularly pronounced in response to the challenges posed by the COVID-19 pandemic [12], necessitating innovative approaches to patient care. Historically, efforts in remote monitoring were primarily focused on non-invasive methods, centered around surveillance of vital parameters such as weight, blood pressure, and heart rate. Subsequent advancements led to the development of cardiac implantable electronic devices capable of remote monitoring based on physiological parameters like intrathoracic impedance [13]. Over the years, multiple trials [14,15,16] have explored various devices for intracardiac filling pressure measurements to monitor heart failure decompensation, with the COMPASS-HF trial [17] notably identifying end-diastolic artery pressure (ePAD) as a surrogate for right ventricular pressure, deepening our understanding of heart failure pathophysiology and suggesting continuous monitoring of pulmonary artery pressure as a potential indicator for effective management before symptomatic acute decompensated heart failure develops. Awaiting evolution of current medical treatments, early detection of hemodynamic changes is crucial for optimizing HF management and improving patient outcomes. This concept encompasses the role of remote monitoring and of telemedicine [10,11], in which sensor technology has emerged as a promising tool. The justification for hemodynamic monitoring primarily stems from the understanding that hemodynamic congestion typically appears weeks before the manifestation of clinical signs and symptoms of heart failure [18,19]. The CardioMEMS™ HF system (Abbott, Inc. Atlanta, GA, USA) serves as a microelectromechanical sensor designed to assess pulmonary artery pressures, and detect changes related to fluid overload in a timely manner. Expanding clinical research supports the effectiveness and safety of CardioMEMS [20,21,22,23,24], and the ongoing German PASSPORT-HF trial is currently investigating the role of these microelectromechanical sensors in patients with heart failure classified as New York Heart Association class III (NYHA), aiming to enhance outcomes and monitor this subgroup of patients more effectively.

## 2. Microelectromechanical Systems

Microelectromechanical systems, known as MEMS, represent a new technology characterized by the miniaturization of mechanical and electromechanical elements through microfabrication techniques [25]. These elements encompass devices and structures with critical physical dimensions ranging from below one micron to several millimeters. The nomenclature for MEMS varies across regions; while they are predominantly referred to as MEMS in the United States, other parts of the world may use terms such as “Microsystems Technology” or “micromachined devices”. Among these, microsensors and microactuators stand out as transducers—devices designed to convert energy from one form to another [26]. Microsensors, in particular, play a pivotal role by converting measured mechanical signals into electrical signals, thereby facilitating the collection and interpretation of crucial data. In recent decades, the field of MEMS has witnessed remarkable progress in the development of microsensors, spanning a wide range of sensing modalities, including temperature, pressure, inertial forces, chemical species, magnetic fields, radiation, and beyond [25,26]. Notably, many of these micromachined sensors have exhibited performances surpassing those of their macroscopic counterparts. This phenomenon is particularly evident in instances such as in pressure transducers, where the micromachined versions consistently demonstrate superior performance compared to sensors fabricated using traditional macroscale machining techniques [27,28]. In light of these advancements, the growing value and impact of MEMS technology in both scientific research and practical applications, particularly within the medical field, are becoming increasingly evident [29]. The true potential of MEMS emerges when miniaturized sensors, actuators, and structures are seamlessly integrated onto a common silicon substrate alongside integrated circuits, thereby forming a cohesive system of microelectronics. For instance, devices like CardioMEMS, designed for measuring pulmonary artery pressure, exemplify this integration.

## 3. State of the Art of MEMS in Heart Failure

The most notable evolution in heart failure monitoring, allowing close follow-up of out-of-hospital patients, has been the emergence of invasive sensors designed to measure intracardiac filling pressures or their surrogates [30]. The rationale behind this approach lies in the belief that hemodynamic congestion precedes clinical congestion, offering a window of opportunity for intervention to avert hospitalizations. The initial implantable sensors were designed for right-sided placement to assess both right ventricular (RV) systolic and diastolic pressures, serving as surrogates for pulmonary capillary wedge pressure and left ventricular (LV) diastolic pressure. Among these, the Chronicle IHM (Medtronic, Inc., Minneapolis, MN, USA) [31] was developed and tested, but was found incompatible with cardiac resynchronization therapy (CRT) devices, which are nowadays standard in managing heart failure patients with reduced ejection fraction. To investigate whether right-sided sensors could effectively predict changes in systolic and diastolic pressures and potentially reduce hospitalizations, the COMPASS-HF trial (Chronicle Offers Management to Patients with Advanced Signs and Symptoms of Heart Failure) [17] was conducted. This trial aimed to assess the clinical impact of hemodynamic pulmonary artery monitoring using the Chronicle sensor in patients already receiving optimal medical care. Although the trial did not achieve its primary clinical goal of demonstrating a statistically significant reduction in heart failure-related events among actively monitored patients, it suggested the potential benefit of such monitoring, prompting further research and development of micromechanical sensors in this patient population. Unlike the Chronicle IHM, which estimated diastolic pulmonary artery (PA) pressures indirectly by measuring RV pressures during pulmonic valve opening, the CardioMEMS HF system was developed to directly measure PA pressures. Currently, CardioMEMS is the only PA pressure sensor approved for routine clinical use with both U.S. Food and Drug Administration (FDA) clearance and European Conformity (CE) mark. Another similar device, the CordellaTM Pulmonary Artery Pressure Sensor System (Endotronix, Inc., Chicago, IL, USA) [32], is also capable of remotely measuring PA pressures and is under investigation. The CordellaTM sensor operates on similar hemodynamic principles as CardioMEMS but is integrated with the CordellaTM Heart Failure System (CHFS), offering additional vital parameter monitoring such as blood pressure, heart rate, weight, and oxygen saturation. Despite its promising features, the Cordella Sensor lacks FDA clearance and the CE mark, remaining under investigation in two clinical studies [33] and not yet available for commercial use. Although evidence supports the utility of PA pressure monitoring, there are scenarios where remote monitoring may not be optimal. Ideally, therapy targeting pulmonary capillary wedge pressure or left atrial pressure would provide more precise management in certain cases. Ongoing research is exploring sensors capable of measuring left atrial pressures (LAPs) to further refine HF management strategies. One such device is the HeartPOD (HeartPOD System, Abbott, Inc., Atlanta, GA, USA) [34], which consists of an implantable sensor lead coupled to a subcutaneous antenna coil. The feasibility, safety, and accuracy of ambulatory hemodynamic monitoring using HeartPOD were suggested in a small clinical study comprising eight patients [35]. Subsequent trials, such as the Hemodynamically Guided Home Self-Therapy in Severe Heart Failure Patients (HOMEOSTASIS) trial, further validated the efficacy of HeartPOD in optimizing left atrial pressures and clinical status in patients with chronic HF in NYHA class III or IV [36]. Additionally, the LAPTOP-HF (Left Atrial Pressure Monitoring to Optimize Heart Failure Therapy) trial [37] aimed to assess the safety and effectiveness of LAP monitoring compared to optimal medical therapy alone. Although terminated early due to an excess of procedure-related complications, the trial provided valuable insights into the potential of LAP-guided hemodynamic monitoring. These findings have spurred the development of the V-LAPTM System (Vectorious Medical Technologies, Tel Aviv, Israel), a more advanced LAP sensor, with ongoing trials such as the VECTOR-HF (V-LAPTM Left Atrium Monitoring System for Patients With Chronic Systolic and Diastolic Congestive Heart Failure) trial [38] evaluating its safety, usability, and performance. While the evidence supporting pulmonary artery pressure (PAP) monitoring is robust, scenarios exist where direct measurement of LAP may offer additional insights, particularly in patients with primary pulmonary disease or elevated pulmonary vascular resistance. Despite promising findings, challenges persist, particularly regarding the safety and usability of left-sided invasive sensors, highlighting the importance of ongoing research efforts to address these concerns. Among the right-sided invasive sensors, the CardioMEMS HF system has emerged as a frontrunner in this domain, demonstrating both safety and efficacy in preventing HF-related hospital admissions.

## 4. CardioMEMS

The CardioMEMS™ HF system Abbott device is an FDA-approved implantable sensor system designed for remote monitoring of PA pressure in HF patients. The system consists of a miniature wireless sensor, implanted in the distal PA via a minimally invasive procedure, and an external Patient Electronics System (PES) for data transmission and display.

**Figure 1:**
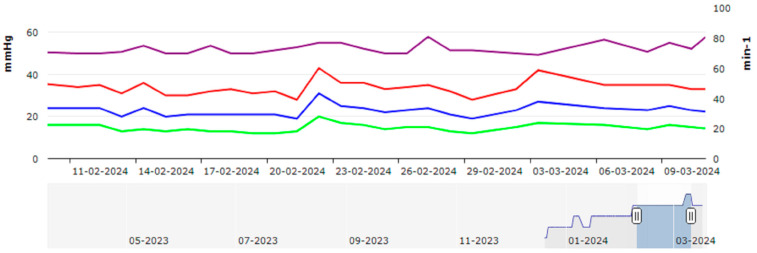
Diagram that illustrates the daily measurements over one month (February to March 2024) from a patient with a CardioMEMS device: computer-stored data showing daily measurements of heart rate (violet), systolic pulmonary artery pressure (red), mean pulmonary artery pressure (blue), and diastolic pulmonary artery pressure (green). Arterial pressure is shown on the left *Y*-axis in mmHg, while heart rate is displayed on the right *Y*-axis in beats/min. The daily measurements are shown on the *X*-axis. These measurements, accessible via secure telemonitoring, provide valuable insights for physicians overseeing the patient’s care. Below the graphic, a continuous blue line represents the average trend of the pulmonary pressure curves since implantation. The area under the curve is depicted either in light blue (periods of time not shown in the detailed graphic displaying the pressure curves) or in dark blue, indicating the time frame represented by the detailed pressure graphic.

The CardioMEMS™ HF system comprises proprietary sensors employing radiofrequency technology for communication. These sensors are implanted using established catheter deployment techniques and are intended to remain implanted for the duration of the patient’s life. Notably, the system does not incorporate leads, generators, or batteries necessitating replacement. Once implanted, the CardioMEMS™ PA Sensor allows the collection of pressure data, which can be transmitted to the treating physician for review and appropriate intervention. The information provided by the HF system encompasses a comprehensive array of parameters, including PA pressure waveform, systolic, diastolic, and mean PA pressure, and heart rate. Patients are instructed to perform self-measurements daily, and these data are subsequently transmitted to a secure online platform. Pressure readings are presented on a user-friendly web-based interface accessible to clinicians for review. Furthermore, the platform is equipped with automated notification features, alerting clinicians in the event of pressure readings falling outside of predefined goal ranges or thresholds, tailored to each individual patient. Clinicians, armed with these real-time data, can make informed decisions regarding medication adjustments and communicate directly with patients as necessary (Figure 1).

## 5. Technical Details

**Figure 2:**
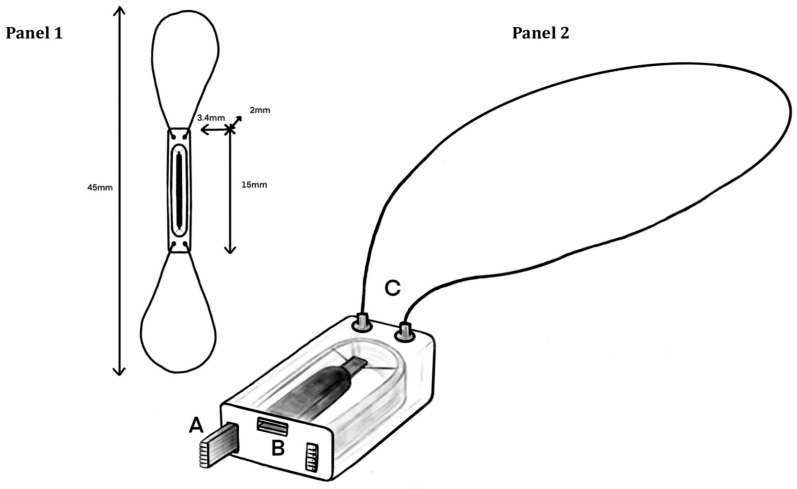
Technical drawing of the CardioMEMS device in its entirety (Panel 1) and a second image showing it in cross-section (Panel 2). Panel 1 displays the device along its longitudinal axis to show length, width, and depth. Panel 2 shows the cross-section image of the device and its components: A: inductor; B: pressure-sensitive capacitor. The capacitor consists of two conductive segments separated by an air gap. Application of pressure on these segments deforms the outer polyimide sheets, bringing the conductive segments closer together and reducing the air gap and consequently changing the capacitance of the circuit. This leads to a shift in the circuit’s resonant frequency, which is directly proportional to the force applied on the sensor’s surface. C: one of the two loops of Nitinol wire extending from each end to allow stability of the device during the implantation. The line art is reproduced with permission from Guido De Filippo.

The CardioMEMS™ HF system comprises a delivery catheter, a hermetically sealed implantable wireless sensor, and an electronic system for data transmission and analysis. At the core of the system lies the wireless sensor, designed for permanent implantation into the distal PA. The PA Sensor comprises a three-dimensional coil (inductor) and a pressure-sensitive capacitor, both housed within two wafers of fused silica measuring 15 × 3.4 × 2 mm. This assembly is enveloped in medical-grade silicone for enhanced durability and biocompatibility (Figure 2). The sensor demonstrates electrical behavior of an LC (“inductance” (L) and “capacitance” (C)) circuit. In this circuit, when a current flows through, energy oscillates between the inductor and the capacitor, leading to an oscillation at a specific frequency known as the resonant frequency. This frequency is determined by the circuit’s inductance and capacitance, which means that any alteration in capacitance will cause a proportional shift in frequency, either upward or downward. The capacitance of the circuit, in turn, changes in relation to the force applied on the sensor’s surface, for example with the blood pressure in the vessel where the sensor is implanted. The inclusion of an inductor enables electromagnetic coupling with the pressure-sensitive capacitor, allowing for wireless communication with a readout device without the need for an internal power source such as a battery. The functional components of the readout hardware (Electronics System) are an antenna and an electronics unit. The antenna sends a radio frequency signal to power the sensor and measures the frequency of the signal returned by the sensor itself. The first calibration of the device is performed in the cardiac catheterization laboratory (Cath Lab) using the blood pressure values obtained invasively from the catheterization of the PA. This initial calibration is necessary to enable subsequent measurements by allowing the resonant frequency received by the electronics unit to be converted into a precise blood pressure value. This signal is processed and stored by the electronics unit. The PES is pillow-shaped and wireless communication with the implanted sensor is established when the patient leans on the pillow for a few seconds and initiates the coupling of the sensor with the ES using a dedicated remote control. Implantation of the sensor is achieved through the use of a transvenous catheter, positioning it within a descending branch of either the left (preferable) or right PA. Nitinol wire loops extending from the pressure sensor play a critical role in securing the implant within a PA branch of significantly larger diameter than the sensor itself. Tether wires serve to connect the PA Sensor to the delivery system until the physician confirms proper positioning within the distal PA. Once confirmed, the tether wires are retracted, freeing the sensor within its designated location.

## 6. Wireless Data Transmission

As previously described, the sensor’s three-dimensional coil facilitates wireless communication with the external Electronics System (ES). Through electromagnetic coupling, the sensor transmits resonant frequency variations to the ES, eliminating the need for an onboard battery. The ES converts these frequency changes into actionable pressure measurements, facilitating seamless data transmission and analysis. The monitoring system comprises the Hospital Electronics System (HES), the PES, and the associated sterile PA Sensor and delivery system. While the HES is utilized within hospital or clinic settings, the PES facilitates home patient monitoring. Although the two systems share similarities, the hospital system is used for the first device calibration and offers enhanced functionality, including the ability to display and print pressure data, a feature not available on the patient version.

The software integrated into the HES enables visualization of pressure measurements on a touchscreen interface during sensor implantation, presenting systolic, diastolic, and mean PA pressure readings along with a waveform. Conversely, the PES software guides and prompts the patient through the process of acquiring a PA pressure measurement, automatically uploading the obtained data to the database. Physicians access patient data through a secure website, which grants them the ability to utilize PA pressure measurements in heart failure management. When patients are hospitalized or present at the clinic/office, the HES facilitates the acquisition of PA pressure measurements, providing physicians with access not only to pressure data but also to accompanying waveforms. Conversely, upon returning home, the PES enables patients to obtain and transmit PA pressure measurements to the database for physician review and analysis [39].

## 7. Implant Procedure

**Figure 3:**
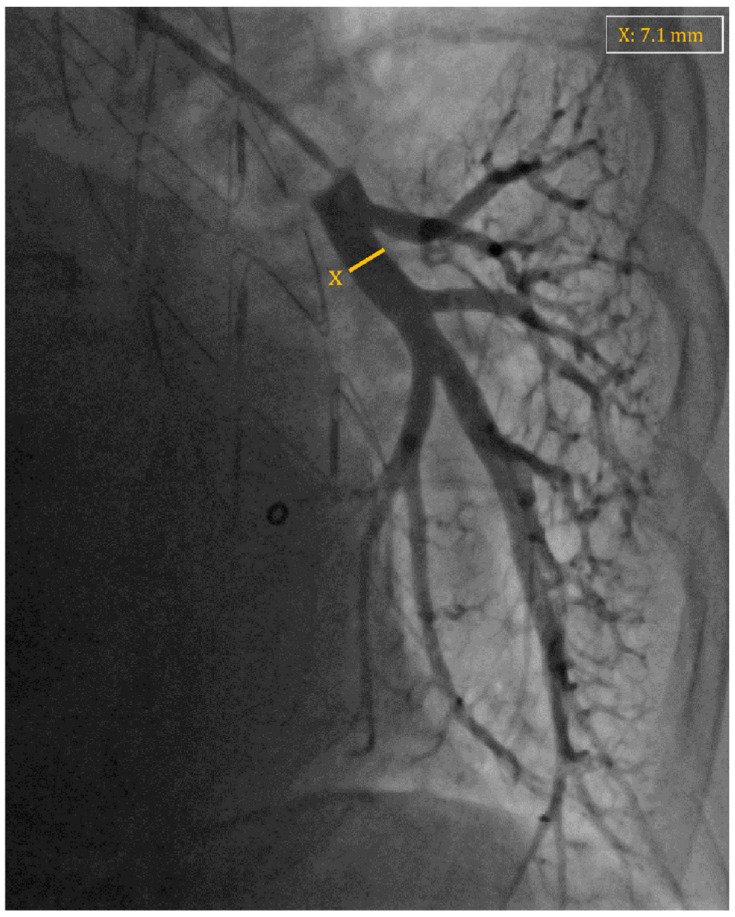
Fluoroscopy image displaying arteriography of a descending branch of the left pulmonary artery chosen as an implantation site for a CardioMEMS device, with a measured diameter of 7.1 mm (highlighted in orange X), meeting the criteria for successful device placement.

**Figure 4:**
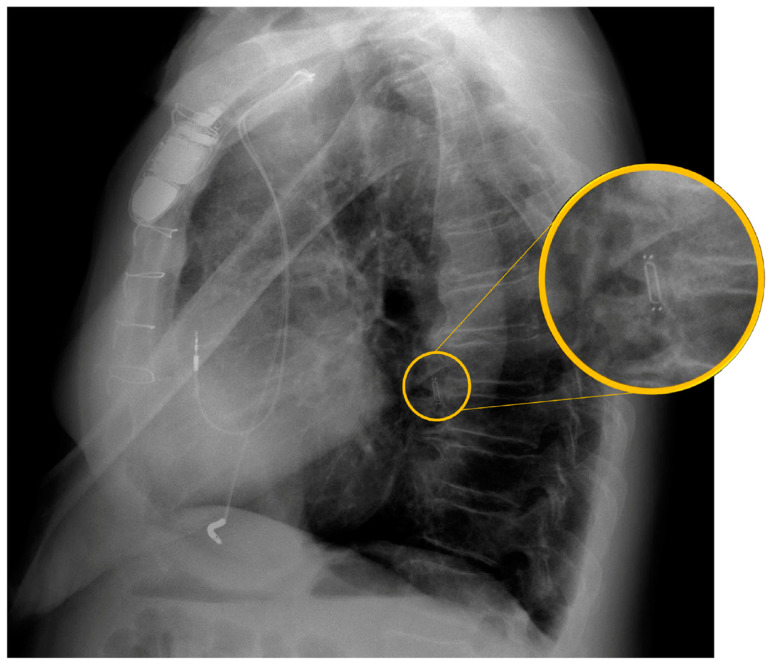
Lateral view X-ray of a patient with a dual chamber implantable cardioverter defibrillator (ICD), showing an implanted CardioMEMS device within a branch of the pulmonary artery. The CardioMEMS device is highlighted in orange, with a zoomed-in view for clarity.

The implant procedure is performed under fluoroscopy guidance in the Cath Lab. The patient is usually awake to achieve maximal compliance for the breathing maneuver during diagnostic right heart catheterization, but in some cases, a small dose of a sedative drug may be administered. The right femoral vein is usually the vascular access of choice, where a 12 French sheath is inserted under local anesthesia, although the internal jugular vein is a possible alternative. A pulmonary catheter (typically a Swan–Ganz catheter) is advanced to perform a diagnostic right heart catheterization, enabling the measurement of right-sided cardiac pressures and the calculation of cardiac output. Afterward, the catheter engages a descending branch of the left pulmonary artery and is used to perform a diagnostic angiogram to assess the vascular anatomy in order to select the implant target site. The target vessel should have a diameter greater than 7 mm with an angulation of less than 30° in the position where the sensor body is to be implanted (Figure 3). A 0.018″ guidewire is introduced through the pulmonary catheter and advanced a few centimeters distal to the catheter’s tip. The tip of the guidewire should always remain straight and preferably avoid prolonged contact or pressure with the distal part of the artery to prevent the risk of vessel perforation. The catheter is retrieved under fluoroscopy, and the delivery system with the sensor is prepared by swirling it in a saline solution for 10–15 s to activate its hydrophilic coating. The delivery catheter is then inserted over the wire through the sheath and carefully advanced to the implant site, while controlling the distal end of the guidewire under fluoroscopy. Once in the target position, the sensor can be deployed by unscrewing and removing the blue cap on the delivery system, and then withdrawing the tether release system. The nitinol wire loops extending from the pressure sensor play a critical role in allowing stability of the device during the implantation in the vessel. Furthermore, platinum/iridium marker bands positioned at both ends of the sensor (a total of four marker bands) facilitate visualization of the device under fluoroscopy during the implantation procedure and subsequent imaging during follow-up assessments (Figure 4). Under fluoroscopy, the delivery catheter is gently withdrawn, leaving the guidewire in place, followed by the reinsertion of the pulmonary catheter over the guidewire to reach the main pulmonary artery, ensuring the sensor remains in position. After the withdrawal of the guidewire, the pulmonary catheter is positioned in the pulmonary artery to measure the pulmonary artery pressure again and calibrate the sensor. The antenna of the HES is placed beneath the patient’s back, and calibration can be performed. At the conclusion of the procedure, the catheter is withdrawn, and the sheath is removed. Usually, suture-based hemostasis is performed, while manual compression or device-based hemostasis are possible alternatives [40].

## 8. Described Complications

The device has undergone rigorous evaluation in clinical trials and postmarketing surveillance to assess its safety profile. Notably, findings from the CHAMPION HF trial [20], the pivotal American multicentric study, played a crucial role in obtaining FDA approval for its use in the USA. In the CHAMPION trial, which included 575 implant attempts, the safety profile of the CardioMEMS device was established. Among the documented serious adverse events were groin hematomas (2 cases), epistaxis (2), hemoptysis (1), unplanned/prolonged hospitalizations (3), recurrence of atrial arrhythmia (2), fever (2), pulmonary thrombosis (1), cardiogenic shock (1), chest pain (1), and delivery system failure requiring intervention (1). Postmarketing surveillance in the USA, as reported in the Manufacturer and User Facility Device Experience (MAUDE) [41] database, further provided insights into real-world safety outcomes. Over the initial three years following FDA approval, more than 5500 CardioMEMS HF system implants were performed across the United States. Among the documented complications were pulmonary artery injury/hemoptysis (0.5%), sensor malfunction, failure, or displacement (observed in 46 cases), bleeding/infection at the access site (15 instances), and pulmonary embolism/device thrombosis (5 reports). Additionally, 22 deaths were reported (approximately 0.4%), 12 of which were of unknown cause or likely unrelated, while some were associated with specific complications such as pulmonary artery injury/hemoptysis and heart failure. Furthermore, findings from the MONITOR HF Study [24], a European randomized multicentric trial involving 176 CardioMEMS implantations, highlighted a high degree of sensor reliability (98.8% freedom from sensor failures). However, device-related complications were observed in four patients (2%), including instances of hemoptysis and arrhythmias requiring intervention. These data underscore the importance of thorough patient selection, operator training, and technological support to minimize the risk of serious complications associated with CardioMEMS device implantation. While adverse events are relatively rare, vigilant monitoring and management strategies remain paramount for optimizing patient outcomes.

## 9. Special Circumstances

Pacemakers (PMs), implantable cardioverter defibrillators (ICDs), and ventricular assist devices are compatible with the PA Sensor and do not interfere with the system’s performance. Furthermore, various medical procedures can be safely conducted with the sensor implanted, provided precautions are taken to prevent direct contact with the sensor. These procedures encompass radiofrequency ablation, ionizing radiation exposure, and diagnostic ultrasound. However, the impact of therapeutic ultrasound on the sensor has not been conclusively determined. Therefore, it is recommended to avoid direct contact with the sensor if therapeutic ultrasound is deemed necessary. If implantation of both a CardioMEMS device and a pacemaker/defibrillator with a right ventricular lead is planned, we suggest proceeding with the CardioMEMS implantation first, if the clinical conditions allow, or waiting for at least 12 weeks after the ICD/PM implantation. This waiting period aims to achieve a more stable lead position and to reduce the risk of lead dislocation or entanglement of the CardioMEMS device during the delivery procedure.

Non-clinical assessments have confirmed that the sensor is magnetic resonance (MR) conditional, meaning it can be safely scanned using magnetic resonance imaging (MRI) immediately after implantation, subject to specific conditions:

- The MRI system must generate a static magnetic field of either 1.5 or 3.0 Tesla.
- The maximum spatial gradient magnetic field should not exceed 720 Gauss/cm (7200 mT/m).

During non-clinical testing, the CardioMEMS PA Sensor underwent MRI scanning for 15 min per pulse sequence in both 1.5-Tesla/64-MHz and 3-Tesla/128-MHz MRI systems. The resulting temperature changes were found to be within safe limits and do not pose any hazard to the patient.

The quality of MR images might be affected if the region of interest overlaps with or is near the location of the sensor. Therefore, it may be necessary to adjust MR imaging parameters to achieve optimal results in such cases. It is important to note that the maximum size of the artifact, as observed on the gradient echo pulse sequence, extends approximately 5 mm from the dimensions and configuration of the sensor.

## 10. Clinical Evidence

Clinical evidence supporting safety and efficacy has been established through several key trials, each offering unique insights into the device’s utility across diverse patient populations and clinical settings. However, even if evidence from the currently available randomized data demonstrates the benefits specifically for the adult heart failure population experiencing symptoms corresponding to NYHA class III or IV, irrespective of the etiology of heart failure, it is important to note that some subgroups, such as women and patients with preserved left ventricular ejection fraction, were underrepresented in certain randomized trials like CHAMPION and MONITOR-HF [42].

The CHAMPION (CardioMEMS Heart Sensor Allows Monitoring of Pressure to Improve Outcomes in NYHA Class III Heart Failure Patients) [20] trial was a prospective, multicenter, single-blinded pivotal randomized, controlled trial evaluating the efficacy of CardioMEMS-guided therapy in reducing heart failure hospitalizations. The trial enrolled 550 subjects with NYHA functional class III HF across 64 sites in the United States. Patients who had a history of HF for at least 3 months with both systolic and diastolic HF were included. Patients with systolic HF (left ventricular ejection fraction [LVEF] < 40%) were included if they were on stable American Heart Association (AHA)/American College of Cardiology (ACC) guideline-based medical and device therapies. Eligible patients also had a history of decompensated HF requiring hospitalization within the 12 months before enrollment. Exclusion criteria included a history of recurrent pulmonary embolism or deep venous thromboses, cardiac resynchronization therapy implantation within the preceding 3 months, and stage IV or V chronic kidney disease (glomerular filtration rate < 25 mL/min). All enrolled subjects received the CardioMEMS HF sensor implant and were randomized to either the treatment group, receiving hemodynamic-guided HF management, or the control group, receiving traditional HF disease management. The trial demonstrated a significant reduction in heart failure hospitalizations in patients who received CardioMEMS-guided therapy compared to standard care. This landmark trial provided robust evidence supporting the safety and efficacy of CardioMEMS monitoring in improving heart failure outcomes.

The GUIDE-HF (Guiding Evidence Based Therapy Using Biomarker Intensified Treatment in Heart Failure) [22] trial evaluated the impact of CardioMEMS-guided therapy on heart failure outcomes in a broader population, including patients with a lower NYHA functional class. This multicenter randomized, single-blinded controlled trial provided evidence of the efficacy of CardioMEMS monitoring in reducing heart failure hospitalizations across diverse patient populations. Patients with chronic heart failure, regardless of left ventricular ejection fraction, NYHA functional classes II–IV, and either a recent heart failure hospitalization or elevated natriuretic peptides, were randomly assigned to either hemodynamic-guided heart failure management based on pulmonary artery pressure or a usual care control group. The primary endpoint was a composite of all-cause mortality and total heart failure events at 12 months. Safety was also assessed. The trial enrolled patients with NYHA functional classes II–IV HF, regardless of left ventricular ejection fraction, with an HF hospitalization within the 12 months before study consent or elevated natriuretic peptides within 30 days before study consent. Patients likely to receive a heart transplant or left ventricular assist device in the next 12 months, patients with advanced heart failure, and those who required inotropes within the past 6 months were excluded. After successful pulmonary artery pressure sensor implantation, patients were randomly assigned to the treatment group (pulmonary artery pressure-guided patient management and standard-of-care guideline-recommended medical therapy) or the control group (standard-of-care guideline-recommended medical therapy only). Patients were masked to their study group assignment, and investigators were aware of treatment assignment but did not have access to pulmonary artery pressure data for control patients. Patient contacts were maintained by designated masked personnel at each site, ensuring balanced communication between groups. Standard heart failure management could incorporate typical data including daily weights, symptoms, and other diagnostics from implantable therapy devices, if available. Patient adherence to daily pulmonary artery pressure uploads was monitored, with poor adherence addressed through scripted reminders by masked callers. This trial did not demonstrate a clear benefit of CardioMEMS implantation based on its primary endpoint, which encompassed all-cause mortality and total HF events, including HF hospitalizations and urgent HF hospital visits. However, it is important to note that the emergence of the COVID-19 pandemic during the trial’s follow-up phase raised unique considerations. To address this, a pre-specified pre-COVID-19 sensitivity analysis was conducted, focusing on results before the pandemic’s onset. Interestingly, during this pre-pandemic period, there was a notable reduction in the risk of the primary endpoint in the active treatment group, primarily attributed to a decrease in the heart failure event rate. Following FDA approval in 2014 for clinical use in the United States, the CardioMEMS Post-Approval Study [23] assessed the real-world effectiveness and safety of the device in routine clinical practice. This was a multicenter, prospective, open-label, observational, single-arm trial conducted across 104 centers in the United States. It enrolled 1200 patients with NYHA class III heart failure and a prior HF hospitalization within 12 months who underwent pulmonary artery pressure sensor implantation between 1 September 2014, and 11 October 2017. The primary efficacy outcome assessed the difference in rates of adjudicated heart failure hospitalizations 1 year after compared with 1 year before sensor implantation. Safety endpoints included freedom from device- or system-related complications at 2 years and freedom from pressure sensor failure at 2 years. Patients with chronic heart failure, regardless of ejection fraction, and NYHA class III symptoms, were eligible for participation, with specific criteria regarding medication usage and body mass index. Key exclusion criteria encompassed factors such as active infection, history of recurrent pulmonary embolism or deep vein thrombosis, recent major cardiovascular events, and anticipated need for heart transplantation or a surgical ventricular assist device within the next 6 months. Implants were attempted in 1214 patients, with unsuccessful pressure sensor implantation occurring in 14 patients. Follow-up visits were conducted at 6 months and 12 months, with the final patient completing 1-year follow-up in October 2018. This observational study provided valuable insights into the use of CardioMEMS in diverse clinical settings, confirming its efficacy in reducing heart failure hospitalizations and improving patient outcomes outside the controlled environment of clinical trials. It demonstrated the translational impact of CardioMEMS monitoring in improving heart failure management in real-world clinical settings. The European MONITOR-HF trial [24] assessed the impact of hemodynamic monitoring of pulmonary artery pressure in patients with chronic heart failure, emphasizing those receiving contemporary guideline-directed medical therapy in the Netherlands. This open-label, randomized trial included 348 patients with New York Heart Association class III heart failure and a history of heart failure hospitalization, irrespective of ejection fraction. Patients were randomized to receive either hemodynamic monitoring using the CardioMEMS-HF system or standard care. The primary endpoint was the mean difference in the Kansas City Cardiomyopathy Questionnaire (KCCQ) overall summary score at 12 months. At 12 months, patients in the CardioMEMS-HF group demonstrated a significantly higher mean change in the KCCQ overall summary score compared to those receiving standard care (7.13 points; *p* = 0.013). Moreover, the CardioMEMS-HF group exhibited higher odds of improvement in KCCQ score (OR 1.69; *p* = 0.046) and lower odds of deterioration (OR 0.45; *p* = 0.0035) compared to the standard care group. Device-related or system-related complications and sensor failure rates were low in both groups. Overall, hemodynamic monitoring using the CardioMEMS-HF system substantially improved quality of life and reduced heart failure hospitalizations in patients with moderate-to-severe heart failure treated according to contemporary guidelines. These findings contribute to the growing evidence supporting remote pulmonary artery pressure monitoring and may impact guideline recommendations and the implementation of this technology in clinical practice.

The ongoing German study PASSPORT-HF trial aims to evaluate the efficacy of a hemodynamic-guided HF nurse-led care approach using the CardioMEMS™ HF system on clinical endpoints. This prospective, randomized, open, multicenter trial is commissioned by the German Federal Joint Committee to assess the effectiveness of PA pressure-guided remote care in the German healthcare system. The trial includes adult HF patients in NYHA functional class III who have experienced an HF-related hospitalization within the last 12 months. Patients with reduced ejection fraction must be on stable guideline-directed pharmacotherapy. Patients are being randomized centrally 1:1 to receive implantation of a CardioMEMS™ sensor or control. All patients will receive post-discharge support facilitated by trained HF nurses providing structured telephone-based care. The trial aims to enroll 554 patients at about 50 study sites. The primary endpoint is a composite of the number of unplanned HF-related re-hospitalizations or all-cause death after 12 months of follow-up, with all events centrally adjudicated. Secondary endpoints include device/system-related complications, components of the primary endpoint, days alive and out of hospital, disease-specific and generic health-related quality of life, and laboratory parameters of organ damage and disease progression.

## 11. Discussion

HF management is increasingly relying on advanced technologies to monitor patients’ physiological parameters and guide therapeutic interventions. Among these technologies, MEMS have emerged as promising tools for HF monitoring. MEMS-based devices, such as the CardioMEMS™ HF system, offer unique advantages in tracking key indicators of HF progression and optimizing treatment strategies. In current clinical practice, HF monitoring involves a combination of traditional methods and more advanced technologies. Biomarkers like B-type natriuretic peptide (BNP) are the cornerstone of HF diagnosis and help in risk stratification. Outpatient HF monitoring utilizes remote monitoring of symptoms through devices like smartphones and online applications (such as Cardiogram (Apple Inc., Cupertino, CA, USA) [43], HeartWatch (Apple Inc.) [44], and AliveCor’s KardiaMobile app [45]), as well as telemonitoring programs with phone calls, video chats, or online platforms connecting patients and physicians [46]. Notably, in Europe, wearable devices with integrated sensors (widely known as “wearables”) made their appearance in the European Society of Cardiology (ESC) Guidelines in 2020 in the field of detecting cardiac arrhythmias [47]. Other implantable technologies, such as lung impedance monitoring, as seen in devices like OptiVol (Medtronic), allow for the assessment of pulmonary congestion by measuring changes in thoracic impedance [48]. This type of monitoring has improved HF management but has limitations when it comes to patient selection, as it only encompasses a patient population who already has or needs a cardiac resynchronization therapy (CRT) device. Other limitations include the lack of specificity regarding changes in intrathoracic impedance, which are also observed in conditions other than HF, such as respiratory infection, and the fact that measurements are performed periodically rather than daily [49]. On the other hand, the implantation of a wireless pulmonary artery pressure sensor that provides continuous hemodynamic data offers a more comprehensive understanding of HF status, allowing for early detection of worsening symptoms and timely intervention. One of the key strengths of MEMS-based sensors is their ability to provide real-time data remotely, enabling proactive management of HF outside the clinic setting. Patients can transmit their physiological parameters to healthcare providers, allowing for timely adjustments to medication dosages and treatment plans. Moreover, MEMS-based monitoring offers personalized insights into individual patients’ responses to therapy, facilitating tailored interventions to optimize outcomes. Despite these advantages, MEMS-based monitoring also presents challenges. Implantation of sensors may carry procedural risks, and device-related complications such as infection or sensor malfunction can occur. Additionally, the cost of implantable MEMS devices may limit their accessibility to certain patient populations. In conclusion, MEMS-based sensors represent a significant advancement in HF monitoring, offering continuous, real-time data that can guide personalized treatment strategies. While there are challenges associated with their implementation, the benefits of improved patient outcomes and reduced hospitalizations justify their integration into HF management protocols. As technology continues to evolve, MEMS-based sensors hold promise for revolutionizing the care of patients with HF, ultimately improving their quality of life and prognosis.

## 12. Future Directions

Bio-MEMS (Biomedical-MEMS) are revolutionizing medical care by offering innovative solutions for patient monitoring, diagnostics, and treatment. As MEMS technology continues to evolve, novel applications are emerging, transforming the landscape of healthcare delivery. Miniaturized sensors integrated into surgical instruments provide surgeons with precise feedback, enabling optimal decision-making during procedures [50]. Additionally, MEMS-enabled surgical tools facilitate minimally invasive robotic interventions, reducing surgical trauma and accelerating patient recovery [51]. The integration of MEMS sensors into prosthetic devices opens new possibilities for personalized healthcare and enhanced patient outcomes. Long-term sensors embedded within devices such as hearing implants [52] and retinal implants [53] enable continuous monitoring of device function and patient health.

Among implantable MEMS for heart failure monitoring, CardioMEMS is only the first FDA-approved sensor for ambulatory monitoring of heart failure patients. However, beyond CardioMEMS, there are ongoing developments in other implantable devices for heart failure monitoring. For instance, LV-MEMS is a leadless, batteryless device being tested, measuring 13.9 × 1.6 mm. Animal studies have shown promise, with LV-MEMS implanted in the left ventricular apex of beagle dogs to induce heart failure and monitor key parameters such as contractility and relaxation [54]. Moreover, MEMS technology is expanding beyond implantable devices to include wearable health monitoring solutions. Smartwatches, hearables [55], wristbands, and other wearables equipped with MEMS sensors are becoming increasingly popular for health monitoring purposes. These devices offer a convenient and non-invasive way to monitor patient activity levels and other vital signs. Importantly, the use of wearables has gained recognition in medical guidelines, with the European guidelines since 2020 encouraging their implementation for diagnosing atrial arrhythmias [47]. This underscores the growing acceptance and adoption of wearable MEMS technology in mainstream healthcare practices. As MEMS technology continues to advance, its impact on the future of healthcare is profound. From remote patient monitoring to surgical innovation, long-term sensor integration in prosthetic devices, and rapid diagnostic capabilities, MEMS offer growing opportunities for improving patient care and advancing medical treatment. From a more practical point of view, one cannot forget that the future increase in remote monitoring technologies may encounter several potential challenges such as the difficulties elderly patients may have in the daily use of telemedicine platforms and digital devices. To address this, comprehensive training and support for patients on how to use the devices effectively, technical assistance hotlines, and user-friendly interfaces must be provided. Additionally, patients in rural or underserved areas may face difficulties accessing healthcare resources, including reliable internet connections and specialized healthcare providers, necessitating the expansion of telemedicine infrastructure and alternative means of communication. Privacy and security concerns surrounding health data may also deter patients and physicians from working on telemedicine platforms, highlighting the importance of robust data encryption protocols, strict privacy policies, and patient education on data security measures [56]. Furthermore, reimbursement policies and regulatory requirements for telemedicine services may vary across regions, posing hurdles for widespread adoption [57]. In conclusion, while MEMS hold significant promise for revolutionizing medical care with their potential to enhance personalized and patient-centered care, their widespread adoption may still encounter resistance due to technological, infrastructural, regulatory, and behavioral challenges.

## 13. Conclusions

Microelectromechanical sensors (MEMS) represent a transformative innovation in the medical field, offering new capabilities for monitoring and managing various health conditions. Within the realm of cardiology, the CardioMEMS device stands out as a prime example of the potential of MEMS technology. By addressing the pressing challenges of heart failure, including recurrent exacerbations, high mortality rates, and frequent hospitalizations, the CardioMEMS device provides a novel solution for continuous monitoring and personalized intervention. This microelectromechanical system operates by measuring pulmonary artery pressure in real time, allowing clinicians to gain valuable insights into patients’ hemodynamic status, recognize the early signs of fluid overload before acute cardiac decompensation in a timely manner, and tailor treatment strategies accordingly. Notably, the device achieves this without the need for a battery, utilizing innovative mechanisms for data transmission and interpretation. Looking forward, the future of MEMS in medicine holds great promise, with opportunities for further integration into clinical practice and expansion into novel applications. As MEMS technology advances, it is expected to significantly improve patient care in different medical fields, allowing for better patient-tailored and patient-centric therapies. In cardiology, the CardioMEMS device is helping to enhance heart failure management and outcomes for patients globally.

## Abbreviations and Acronyms

HF	Heart failure
MEMS	Microelectromechanical Systems
Bio-MEMS	Biomedical Microelectromechanical Systems
PA	Pulmonary artery
NYHA	New York Heart Association
ePAD	End-diastolic pulmonary artery pressure
PES	Patient Electronics System
HES	Hospital Electronics System
KCCQ	Kansas City Cardiomyopathy Questionnaire
FDA	Food and Drug Administration
CRT	Cardiac Resynchronization Therapy
Opti Vol	Feature of some implantable cardiac devices used for monitoring changes in intrathoracic impedance. Developed by Medtronic
ESC	European Society of Cardiology
